# Using Process Indicators to Monitor Documentation of Patient-Centred Variables in an Integrated Oncology and Palliative Care Pathway—Results from a Cluster Randomized Trial

**DOI:** 10.3390/cancers13092194

**Published:** 2021-05-03

**Authors:** Marianne Jensen Hjermstad, Julian Hamfjord, Nina Aass, Olav Dajani, Tonje Lundeby, Torunn Wester, Stein Kaasa

**Affiliations:** 1 Department of Oncology, Oslo University Hospital, 4950 Oslo, Norway; UXHAJU@ous-hf.no (J.H.); NAA@ous-hf.no (N.A.); UXOLAJ@ous-hf.no (O.D.); tonje.lundeby@medisin.uio.no (T.L.); TOWEST@ous-hf.no (T.W.); stein.kaasa@medisin.uio.no (S.K.); 2 European Palliative Care Research Centre (PRC), Department of Oncology, Oslo University Hospital, 4956 Oslo, Norway; 3 Institute of Clinical Medicine, University of Oslo, 0318 Oslo, Norway; 4 Faculty of Medicine, University of Oslo, 0372 Oslo, Norway

**Keywords:** trials, palliative care, cancer, patient-centred care, patient pathway, symptom control, implementation science, integration, process evaluation

## Abstract

**Simple Summary:**

The body of research from randomized controlled trials (RCTs) demonstrates that palliative care (PC) alongside anticancer treatment improves patient and caregiver outcomes, e.g., tolerance to treatment, symptom control, and satisfaction with care. This results from integrating the patient-centred focus of PC with the traditional tumour-directed focus in oncology. This integration represents a complex intervention affecting how people work. We have investigated clinicians’ adherence to requested documentation of four important patient-centred study indicators (EGOC; symptom assessment, weight and GP report) in our ongoing RCT on PC integration. Results from 435 consultations; first oncological (start last chemotherapy-line), palliative and oncological consultations during chemotherapy showed that registration percentage differed across consultations; 94.8% in the palliative (83.3–100%), 65.8% (62.5–75.0%) and 69.2% (57.0–84.3%) in the oncological consultations. Results were not satisfactory and call for strict pre-study optimization strategies to promote integration and handle organizational, professional and individual barriers towards a more patient-centred focus.

**Abstract:**

Background. Despite robust evidence from randomized controlled trials (RCTs) demonstrating clinical and patient-reported benefits of integrated oncology and palliative care, the tumour-centred focus is predominant. This single–centre process evaluation monitors documentation of required patient-centred variables during an RCT. Methods. Performance status, patient self-reported symptoms, weight and summaries to general practitioners were assessed from June 2017 to July 2020 in three consultation types: first oncological after study inclusion and palliative and oncological consultations during chemotherapy. Descriptive statistics were used to monitor if the pre-defined program fulfilment of ≥85% documentation was reached. Results. 435 consultations were monitored in 76 patients; 60.5% males, 86.8% with GI cancers; 76 (17.5%) were from the first oncological consultations, 87 (20.0%) and 272 (62.5%) from palliative or subsequent oncological consultations. Program fulfilment differed across consultation types with 94.8% in the palliative consultations (83.3–100%), relative to 65.8% (62.5–75.0%) and 69.2% (57.0–84.3%) for first and subsequent oncological consultations over time, respectively. Use of self-reported symptoms was consistently lower in the oncological consultations. Conclusions. The documentation level of required core variables was not satisfactory, notwithstanding their high clinical relevance and continuous reminders during study. Pre-trial optimization strategies are paramount to promote integration and reduce professional and personal barriers towards a more patient-centred focus.

## 1. Introduction

Palliative care (PC) complements anticancer treatment and may actually improve the effect of this treatment by optimizing performance status, functioning, symptom management and the transfer of information between medical specialties and health levels [1,2]. This is supported by a series of randomized clinical controlled trials (RCTs) investigating early PC alongside anticancer treatment [3,4,5,6,7,8,9,10]. A recent Cochrane Review documented that the statistical evidence on provision of early PC is weak with small effect sizes but concluded that this approach might still be of high clinical relevance to patients approaching end-of-life (EoL) [11].

The patient-centred approach is the mainstay of palliative cancer care with the primary focus being the patient with the disease [1]. However, the increasing demand for medical treatment and cure in the society at large means that the tumour-centred focus is predominant at the expense of the patient-centred focus, which has led to more intensive treatment at the end of life, often with marginal effects on survival and patient wellbeing [1]. Moreover, patient and caregivers’ preferences and understanding of prognosis and stop criteria are essential in anticancer treatment, particularly so when given with a life-prolonging intent [12]. This contrasts the WHO palliative care definition of “… impeccable assessment and treatment of pain and other problems...” from early on in a disease trajectory and in conjunction with other therapies [13]. This corresponds to the Institute of Medicine (IOM) definition of patient-centred care stating that patient values and preferences should guide clinical decisions [14], in line with clinical guidelines from the American Society of Clinical Oncology (ASCO) and European Society for Medical Oncology (ESMO) stating that patient-centred care is an important part of all clinical cancer care [15,16].

The use of patient-reported outcome measures (PROMs) in research and clinical oncology and palliative care has been advocated for decades, being reinforced by the Food and Drug Administration (FDA) requiring their use in clinical trials for supporting a labelling claim in the medical industry in 2009 [17]. The body of evidence regarding the benefit of using PROMs in a patient-centred context to improve patient outcomes is indisputable, with examples such as better communication between health care providers and patients, raised awareness and better management of symptoms, extended survival time, advising treatment decisions, and informing service delivery [2,18,19,20,21,22,23]. However, the use of PROMs to elicit patients’ views is not as widespread nor as systematic as necessary for provision of good quality care, not in general oncology, nor in palliative care [22,24,25,26]. In fact, reviews and meta-analyses show that 38% of cancer patients report moderate to severe pain (≥5 on 0–10 scales) and that one-third does not receive adequate pain medication [27,28].

It is well known that trial results translate poorly into clinical practice, often because study outcomes do not reflect real-world settings or are not perceived relevant to clinical concerns [29]. Moreover, studies may be compromised by methodological problems in design, conduct and samples that may threaten the external and internal validity of the results. Organizational, institutional and contextual barriers such as a general resistance to change, a tendency to prioritize professional individualism over research findings, and the never-ending demand for extra resources due to expected time constraints are frequently encountered barriers in complex health care organizations [30,31].

We are currently running the PALLiON-study (PALLIative care in Oncology); an ongoing Norwegian cluster-randomized controlled trial (C-RCT) aiming to improve the quality and efficacy of cancer care for patients with advanced cancer who receive chemotherapy [32]. The use of patient care pathways adapted from the European Pathway Association [33] is the bearing element of the intervention cluster and provides an organization of anticancer treatment, patient and caregiver PROMs and follow-up, and integration of oncology and palliative care. The primary study outcome of PALLiON is use of chemotherapy in the last three months of life [32]. The overall, long-term objective is to implement systematic use of PROMs in the entire oncology department at our own institution, Oslo University Hospital (OUH). At present, this is only routine in our palliative care team.

Although theories underpinning implementation science may help to understand why implementation of PROMs in clinical practice has not come further [34,35], conducting an implementation research project amidst a C-RCT is a major endeavour. Thus, we decided to do a small-scale pragmatic process evaluation at OUH, a tertiary cancer referral centre and the main PALLiON study site, to monitor the use and documentation of patient-centred variables during patient inclusion. The intention was to examine how we fared, use the findings to improve study conduct as necessary and subsequently to guide further implementation of PROMs in our clinic. This paper presents the proportion of selected study specific core variables that had been documented in the patient records as required, related activities to improve and lessons learned.

## 2. Methods

### 2.1. The PALLiON Intervention

PALLiON is a parallel group, national, multicentre C-RCT on early provision of palliative care with a complex three-tiered intervention in one arm (six hospitals) and conventional care in the other six. The main inclusion criterion on the patient level is a diagnosis of solid tumours at start of last line of conventional chemotherapy. Further details can be found in the PALLiON protocol paper [32].

The first part of the intervention is a study specific educational program for oncologists, PC physicians and residents. It consists of lectures, discussion groups, communication skills training and coaching [36]. This evidence-based program aims to promote the integration of oncology and PC by reducing barriers to early PC referrals, through enhanced competence and understanding of advanced cancer treatment and specialized PC with a patient-centred focus.

**Figure 1:**
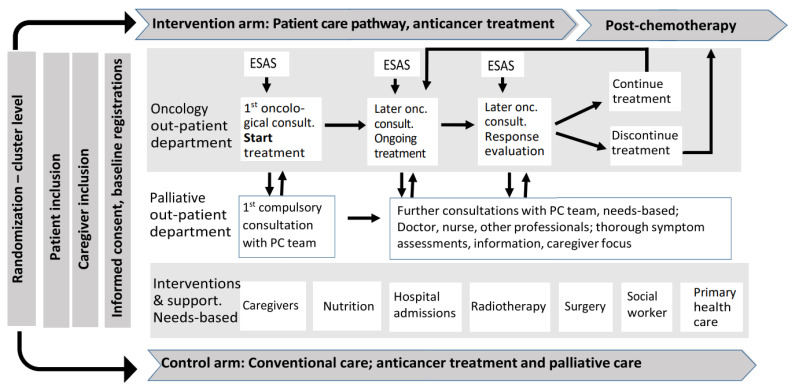
Overview of the PALLiON patient pathway. ESAS; Edmonton Symptom Assessment System [37].

The other two parts of the intervention are embedded in an integrated oncology and palliative care pathway that is implemented in clinical practice. The pathway facilitates the organization of the cancer treatment and care activities from study inclusion through follow-up (Figure 1). The first part has two major elements, (a) systematic, mandatory symptom registrations prior to the patient consultations for immediate use in the pathway, using the Edmonton Symptom Assessment System (ESAS) [37] and (b) monthly postal patient and caregiver self-report forms on symptoms, quality of life (QoL) and satisfaction with care for research use.

All parts of the care pathway were interactive with clinical information and templates for each consultation types, treatment guidelines and links to relevant resources. The study templates comply with national and international treatment guidelines, long-term clinical experience, literature searches and round table discussions. Included elements were deemed essential for the delivery of quality cancer and palliative care in the chemotherapy pathway [32]. The consultation templates provide detailed descriptions of the intended content of the consultations and outline the clinical data for registration. Four core variables were mandatory to document in the patient records at all oncological and PC consultations; ESAS, Eastern Cooperative Oncology Group (ECOG) Performance Status [38], weight plus a written summary to the patient’s general practitioner (GP). Other consultation specific variables to address were nutritional status, symptom burden, side effects and response to treatment, supplemented with more patient-centred variables, e.g., understanding of prognosis and treatment intention and preferences for care including “what’s most important to you now” (Table 1). These variables were listed but not defined as mandatory in the consultation templates. The interactive pathway was easily accessible on all computers within and outside the hospital network by a shortcut key.

### 2.2. The Monitoring Strategy and Procedures

The Medical Research Council (MRC) has developed a guidance that provides a framework for conducting and reporting process evaluations in complex trials [30]. Some of the principles are embedded in our institutional patient safety program using the Plan-Do-Study-Act model (PDSA), developed by Shewart in the 1920s and known as the Deming cycle in organization development programs [39,40].

PDSA is an iterative, four-stage problem-solving model used in different ways to control and improve a process and secure that the desired changes remain stable over time. We selected this model on two grounds; (1) it is often used on a small scale before embarking on larger implementation or change processes and can be used in ongoing studies or improvement projects, and (2) it fits well with the decision that essential clinical and patient-centred data must be addressed and documented routinely in the PALLiON pathways, and preferably also after study closure. Moreover, a large-scale process evaluation amidst the RCT exceeded our capacity.

The basic questions in a PDSA model are; “what do we want to achieve, what are the necessary changes to improve, how to know if a change represents an improvement?” The first question above defines the aim; to investigate if the required data were assessed and documented, while the other two evaluate if the documentation was at a satisfactory level and remained stable over time. The PALLiON management group made a checklist by defining a number of important patient-centred variables from the templates in the pathway. These served as quantifiable process indicators in our PDSA model. The proportion of these was defined as program fulfilment per consultation type. Ideally, one should aim for a 100% completion or fulfilment rate, but this was regarded as unattainable even in a study setting. Thus, we decided that ≥85% indicated a satisfactory program fulfilment, with a change of ±20% prompting the need to maintain or improve.

If improvements were deemed necessary, our pre-planned strategy was implemented. Improvements included, but were not restricted to, repeated and continuous prompting by leaders and study personnel at clinical formal and informal meetings and in pre-round discussions. Posters were hung on the consultation rooms walls. Physicians received pocket-size cards with tables showing what to document at the different consultations and the overview of the pathway as in Figure 1, including the shortcut key to the interactive pathway. Moreover, study specific templates were incorporated in the patient records to facilitate data documentation. In this period, physicians were also enrolled in the communication skills training program that included coaching on their communication with patients.

The following three consultation types were chosen for monitoring; first oncological consultation after study inclusion upon start of anticancer treatment, the compulsory and subsequent PC consultations and oncological consultations during chemotherapy (Figure 1, Table 1). These three consultations represent the three main consultation types and as such the bearing elements of the pathway. Documentation of the four core indicators; ESAS, ECOG, weight and summary to GPs was monitored in all three consultation types, together with the sets of the six, five, and four consultation specific indicators that applied to each of the three consultation types, as listed in Table 1.

Two of the authors (JH/TW) reviewed retrospectively the electronic patient records of PALLiON-patient consultations included at OUH until July 2020 during the following five periods: I: 19 June 2017 to 8 January 2018, II: 9 January 2018 to 31 May 2018, III: 1 June 2018 to 3 January 2019, IV: 4 January 2019 to 7 October 2019 and V: 8 October 2019 to 1 July 2020.

### 2.3. Statistical Considerations

The indicators were assigned a value of 1 if documented and 0 if missing. However, a non-penalizing value of 1 was used for some of the missing consultation specific indicators e.g., documentation of preferences for care, in cases where the patient records showed that this issue had been addressed a few days prior to the actual consultation. This evaluation was at the discretion of the reviewers, who consulted each other in cases of uncertainty.

**Figure 2:**
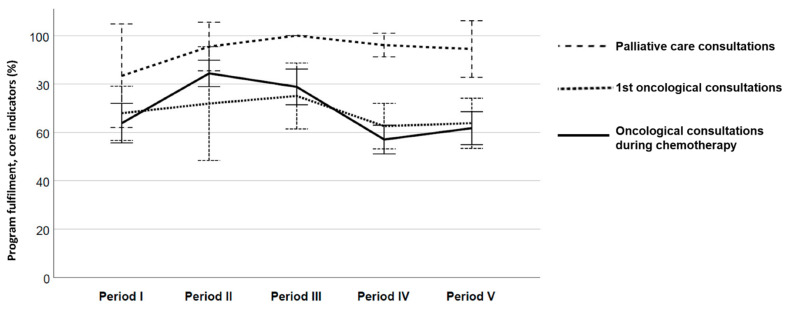
Program fulfilment, core indicators by period and consultation type.

**Figure 3:**
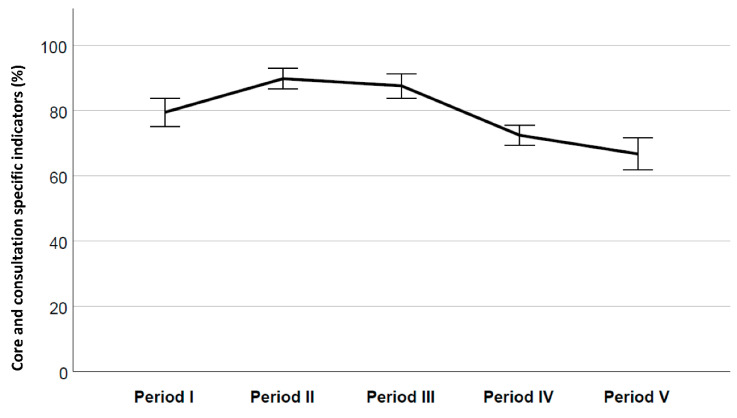
Overall program fulfilment by period, core and consultation specific indicators combined.

Descriptive statistics were used to assess the proportion and percentages of documented indicators for each consultation. Program fulfilment of the core indicators was calculated as the percentage of documented core indicators with a value of 1 relative to the total number of core indicators for documentation. Overall program fulfilment was calculated as the percentage of documented core and extended indicators with a value of 1 relative to the total number of indicators for documentation. A thorough quality assurance of the data file was performed, and if a surprisingly low program fulfilment was revealed, the relevant patient records were reviewed once again. The presentation of the results is as follows:

- Tables showing the documentation of each core indicator by time period for each of the three consultation types (Table 2)
- A figure showing program fulfilment for the core indicators by consultation type for each period (Figure 2)
- A figure showing program fulfilment by period, representing the summary proportion of all consultations and indicators (core and consultation specific) (Figure 3)

Results and trends over time are displayed by modified statistical process control (SPC) charts recommended for process evaluations, useful for examining if there are special reasons for variation over time [41]. Our modification was plotting mean proportions over time, rather than every single consultation independently. Data were plotted in time order, showing the average program fulfilment, supplemented by upper and lower 95% confidence intervals (CI). These figures facilitated the distribution of scores, identified need for improvement in the different consultation types and clearly depicted program fulfilment and deviation from the predefined level of 85%. Statistical software used was IBM SPSS Statistics for Windows, version 26.0, (IBM Corporation, Armonk, NY, USA).

### 2.4. Ethical Considerations

Ethical approval for the PALLiON-study protocol (Dec. 2016 was confirmed by the Regional Committee for Medical and Health Research Ethics, South East Norway (RefID: 2016/1220-PALLiON). No further approval was necessary for the present study, as this is part of the study evaluation process. All patients have signed the study consent forms that include extraction of clinical data from the medical records. The PALLiON trial is registered in the ClinicalTrials.gov database (No. NCT01362816).

## 3. Results

The consultations were conducted in 76 individual patients, of whom 61.6% were males. The majority (86.8%) had GI cancers. 435 consultations were reviewed in the five periods, 76 (17.5%) from the first oncological consultation, 87 (20.0%) from PC consultations and the remaining 272 (62.5%) from oncological consultations during chemotherapy (Table 2).

As can be viewed from the tables, program fulfilment, i.e., proportion of documented core indicators of those required varied across consultation types and periods of monitoring. First, this applies to program fulfilment per consultation type when looking at all periods combined, being 65.8% for the first oncological consultations, relative to 94.8% and 69.2% for the palliative and subsequent oncological consultations, respectively (Table 2). Second, the proportion of documentation varied across periods for all consultation types, although with no consistent pattern of fluctuations (Figure 2). The differences were most pronounced for the oncological consultations during chemotherapy, varying from 57.0% to 84.3%.

Six periods had a program fulfilment above 80%; all in the first PC consultations and one in the oncological consultations during chemotherapy. The predefined level of program fulfilment ≥85% was obtained in four periods in the PC consultations, with slightly lower percentages (83.3%) in the first period here and in the second period for the oncological consultations during chemotherapy (84.3%). Variations across periods are shown in Figure 2.

When looking more closely at each of the core indicators in Table 2, documentation of ECOG and reports to the patients’ GPs are generally high across consultation types while the documentation of weight is more fluctuating. Further, documentation of use of ESAS is considerably lower in the first oncological consultations relative to the PC and subsequent oncological consultations. The surprisingly low rates in the last two periods in the subsequent oncological consultations were verified by double-checking the patient records, but no errors were detected.

As explained, the consultation specific variables other than the core variables (Table 1), were all part of the consultation template, and strongly recommended to assess and document. The summary of all core and patient-centred variables for all consultations was regarded as the overall program fulfilment. Figure 3 shows that two periods (II and III) attained the predefined level of ≥85%, with mean levels of 89.7% and 87.5% respectively, while results for periods IV and V were considerably lower (72.3% and 66.6%) (Figure 3).

## 4. Discussion

Improving clinical care delivery and quality is challenging, primarily because it aims to change how people work by disrupting traditional routines and presenting new systems for interaction and information exchange [42]. The published RCTs on integration of oncology and palliative care have implemented multiple changes in their intervention arms, many that affect the way people work. However, we have not found any publications describing sustainable changes in clinical practice following these RCTs. This is probably because study procedures often come to a halt after study closure, and because identifying the most effective elements of multicomponent interventions is difficult, and calls for specific research strategies [43,44].

Here we present results from a monitoring of the proportion of documentation of four core variables that were required during a cluster RCT [32]. The reason for doing this was to evaluate how we fared during study, perform adjustments as necessary and probably make use of the experience to guide the course towards our long-term objective of integrating tumour- and patient-centred care in our oncology department.

Our results revealed a considerable difference in our predefined program fulfilment. First, the most pronounced difference regarding the four core variables; ESAS, ECOG, weight and written summaries to GPs, was found between the two oncological consultations on the one hand with completion rates of 65.8% and 69.2%%, relative to 94.8% in the palliative care consultations.

Several factors may relate to this. First, the outpatient oncology department is large with a daily average of 49 chemotherapy visits per month in 2019. This means that a substantial number of physicians were involved in the consultations. There was also a relatively high staff turnover during the protracted period of patient inclusion from June 2017 to July 2020, because of the COVID-19 pandemic. Both factors may have led to many physicians feeling little or no ownership to the PALLiON-study, despite continuous information activities and reminders. These assumptions on our part were confirmed when looking at the documentation at the individual physician level, showing considerable differences (data not reported).

Second, the PC department is much smaller, with a handful of physicians being responsible for the consultations during the study period. When looking at the specific core variables, it is encouraging that all but one period had an average documentation rate above 85%. ESAS is routinely used by the PC outpatient team, most certainly influencing the high fulfilment. Moreover, the PALLiON study was initiated by our palliative care research centre (PRC) [45] that may have inferred an obligation and motivation to follow study procedures, also for new residents on rotation. The slight improvement from period I to III in the oncological consultations adds to this, given the intensified prompting by a senior physician in this period, emphasizing that information and reminders are a continuous effort during inclusion and beyond, with a motivated staff being instrumental [1,46].

Patient-centred care is embedded in palliative medicine, also alongside anticancer treatment. The two are integrated and intertwined with patient perspectives on the situation. Oncology practice is predominantly tumour-centred focusing on treatment, be it curative or not [1]. This is probably the most plausible explanation of the relatively large differences between the oncological and PC consultations in terms of documentation, despite the fact that the chosen core indicators are essential clinical variables. The variable documentation rate of patients’ weight in the oncological consultations is thought provoking. The documentation of weight was generally higher in the first oncological consultations than during treatment. We attribute this to weight being an important variable for initial dose calculations of chemotherapy, underlining the tumour-centred focus in these consultations. Importantly however, weight loss and poor appetite are important to patients as well as essential determinants of cachexia, poor tolerance to anticancer treatment and hastened death [47,48], underpinning the importance of this variable.

Documentation of ESAS use fluctuated in the oncological consultations. We have no obvious explanations for this, other than the fact that missing documentation does not necessarily indicate that patients’ symptoms were not attended to. Nevertheless, it is beyond reasonable doubt that the systematic use of a validated instrument for comprehensive symptom assessment was not used as intended in the study, despite oral reminders, wall posters, distributed pocket-size cards and interactive pathways with ESAS displayed on top. Taken together, weight and symptom scores are clinically important variables related to both tumour- and patient-centred perspectives and should be routinely assessed and documented in all consultations. It is disappointing but not surprising, that consistent, robust evidence from RCTs and reviews and international guidelines on the benefits of using PROMs even on survival is not sufficient to change practice [2,16,23,25,49,50]. Apparently more complex strategies are necessary to make the physicians realize the added importance of using PROMs.

The overall program fulfilment when looking at all consultations and all variables combined seemed reasonably good, judging from Figure 3. The consistently high documentation rate in the palliative care consultations contributed to this, but it should also be noted that this combined curve may be somewhat inflated. By reference to the statistical section, a value of 9 was assigned to the consultation specific indicators if these were not deemed relevant in the given consultation, e.g., if referral to specialized PC had already taken place, or adverse effects had been documented the day before. This imputation reduced the proportion of missing, but one could argue that the doubt benefits the accused, in that we cannot ascertain if these issues had actually been discussed with the patient. Interestingly, a recent study showed that the use of patients’ self-report by clinicians differs by their relationship with the patient and their own personal and professional beliefs [51]. In our opinion, this also relates to the autonomy and independence in the way physicians work. Nevertheless, thorough documentation is crucial and it is alarming when up to 80% of patients with advanced stage metastatic lung and GI cancers and malignant gliomas may not realize that their chemotherapy is probably not curative [12,52]. Studies also show that patients who have discussed the future with their doctor more recently or before the disease is too far advanced, have a more realistic understanding [53]. Patient-centred communication enhances patient and family involvement, guides decision making, promotes realistic expectations for the future [54,55], and improves transmit of information and continuity of care, both frequent reasons for patient complaints [56]. The review by Hui et al. [55] emphasizes the need for a patient-centred approach when discussing prognosis to know where the patient is along the prognostic continuum to improve patient outcomes, in line with the review by Back [57].

It is interesting, albeit partly understandable, that the program fulfilment was better for the more objective, clinical core variables than for the consultation specific more patient-centred variables, e.g., understanding of prognosis and the situation. This was substantiated with better documentation rates when looking at the two more objective variables in the oncological consultations during chemotherapy; i.e., specific adverse effects and response to treatment according to established criteria (data not reported). Communication about prognosis and treatment gives rise to physician and patient related barriers alike. One thing is the emotional element on the part of physicians, patient and family that is difficult to handle and relates to fostering hope [58]. Further many physicians experience a psychological discomfort associated with disclosing a poor prognosis and feel they lack the necessary skills to communicate bad news [59]. Although this skill improves by training [60], there is still a way to go. More importantly is probably the predominant tumour-directed focus in oncology implying that stopping treatment may be perceived as failure. However, the most recent ASCO summary of guidelines for patient and clinician communication provides a set of strong recommendations and advice on what to discuss with patients in challenging situations that often are insufficiently adhered to [61], as anticancer treatment is in the forefront.

A traditional assumption has been that presenting convincing evidence-based research results would suffice to an uptake in practice [62]. A Canadian study compared the timeliness of PC referrals defined as early (12 months) or late (<6 months) before death in two patient cohorts, one from 2006 before the published evidence of early PC referrals, and one from 2017 when the positive benefits were well-known and wide-spread demonstrating a substantial increase in early referrals [63]. From an implementation point of view, this approach may still work for relatively small and specific changes but based on our experience from the present study and our former work, a more comprehensive approach is necessary. In complex interventions in particular, it is essential to focus on the context in which the changes will take place including the need to define and approach facilitators and barriers on many levels, consider the professional and social contexts and maybe first, understand the organizational dynamics [64,65]. Health care and hospitals are social systems characterized by interaction among several agents within and between levels, formal and informal social subsystems and rules, in which changing ways of working gives rise to alliances that may cause discontinuance. Moreover, the system history and starting point play an important role [65].

One could argue that early provision of palliative care should take place long before start of the anticipated last line of chemotherapy. However, as this was a multicentre RCT examining potential benefits of systematic palliative care, we wanted to make sure that the intervention had a common starting point for all included patients at the intervention and control sites alike. Notably, patients with an established contact with the palliative care teams were not eligible for the study [32].

A potential limitation may also apply to the definition of anticipated last line of chemotherapy as one may think that this precludes subsequent treatment or participation in pharmacological studies later on. As presented in the study protocol paper [32], anticipated last line was defined according to established Norwegian diagnosis-specific treatment guidelines at the time. For the treating oncologists, anticipated last line as well as estimated survival time for that matter, is a product of prognostication and clinical experience, hence implies some uncertainty. However, PALLiON does also reflect clinical practice, and does not preclude further therapy if treatment guidelines change during study.

Importantly, we have only looked at one element of study conduct in three selected consultation types that may not be representative for the study as a whole. Further, one could argue that numbers are too small to conclude. However, our method was not selected to present statistical evidence related to documentation of patient-centred variables as such, but to address important aspects related to implementation of even small changes. It is reasonable to say that we fell short in certain aspects of the preparatory work and underestimated the amount of necessary pre-study information and continuous, iterative reminders to improve. Clear strategies on top-down leadership and enthusiasm are paramount and we should have recruited more key personnel and opinion leaders as stakeholders from offset, preferably among senior physicians as motivators. A chemotherapy pathway was to be followed, referral to specialized palliative care was mandatory at start of last line of chemotherapy, ESAS was introduced for assessment and documentation of PROMs and study specific variables should be addressed in the different consultation types. Taken together, a complex intervention. The educational program prior to start of patient inclusion was intended for involved physicians. The younger physicians were more eager to participate, more receptive to the content, and more active in discussions and role-plays about breaking bad news and patient-centred communication in the program. Not all physicians completed the entire program, with fewer senior physicians following the communication part. This may have contributed to persisting presuppositions about palliative care referrals, patient-centred care and benefits of integration with oncology that exist both on the part of physicians, other health care providers and patients [66,67,68,69]. Despite the previously mentioned interventions to improve, program fulfilment at the oncological consultations remained disappointingly low. Introducing templates in the patient records with mandatory registrations of the four core variables may have contributed to increase the documentation rate, but this was not an option from a technical point of view.

Taken together, this relates to what we perceive as the most important barrier, namely the dominating tumour-focus in general oncology. This is further reinforced by an internalization of values of autonomy in the work behaviour from medical school and onwards, coupled with an inclination of physicians’ professional individualism and autonomy and perceptions of self-efficacy to manage PC needs [68]. The latter does not correspond with the increasing use of anticancer therapy at end of life [70,71]. Nevertheless, informing patients with advanced disease and a short life expectancy about prognosis, treatment intent and stop criteria are core clinical skills in integrated oncology and palliative care, fundamental in patient-centred care [1], and should be appropriately documented in all consultations. When one does not succeed with relatively small changes in a study setting, a permanent change of clinical practice is a major endeavour on many levels. This may be one reason why publications about sustainable changes in clinical practice in the wake of RCTs on integration of oncology and palliative care are missing.

## 5. Conclusions

Our small-scale process evaluation showed that a small part of study conduct in this complex intervention was not adhered to at a satisfactory level. This was despite of the fact that the selected indicators were essential clinical variables to be documented in routine clinical care. This, once again, shows the challenges in changing the way individual professionals work. Pre-trial optimization strategies with a strict administration of a compulsory educational component, the conduct of pilot or feasibility studies prior to implementation and better identification of potential barriers are crucial. Further, recruiting a sufficient number of senior staff possessing formal and informal influence is key. These factors are relevant to larger and smaller changes in health care, but importantly also to clinical trials, to avoid undesirable bias or suboptimal results.

## Figures and Tables

**Table 1:** Core and consultation specific indicators in the PALLiON pathway consultations.

Process Indicators
Core process indicators in all consultation types (*n* = 4) ^1^
ESAS score ^2^ ECOG score ^3^ Weight Written summary sent to patient’s GP
Consultation specific process indicators; first oncology outpatient consultation (*n* = 6) ^4^
Treatment intention when starting last line of chemotherapy Side effects and management Criteria and reason for discontinuation of chemotherapy (i.e., stop criteria) Referral to palliative out-patient department Patient preferences; what is most important now Patient understanding of the treatment situation
Consultation specific process indicators; palliative care consultations during chemotherapy (*n* = 5) ^4^
Patient preferences; what is most important now Patient understanding of the treatment situation Caregiver issues; relationship and understanding of the situation Plan for symptom management Written summary sent to responsible oncologist
Consultation specific process indicators; subsequent oncology outpatient consultations during chemotherapy (*n* = 4) ^4^
Side effects Response to treatment Needs-based referral to PC outpatient department Compulsory referral to PC outpatient department team upon discontinuation of chemotherapy

^1^ marked as mandatory to address and document in the consultation templates. ^2^ Edmonton Symptom Assessment System [37]. ^3^ Eastern Cooperative Oncology Group Performance Status [38]. ^4^ recommended to address and document in the consultation templates.

**Table 2:** Proportion of documented core indicators and program fulfilment, all consultation types; first oncological, palliative care and oncological during chemotherapy.

**First Oncological Consultations**	**Period I**	**Period II**	**Period III**	**Period IV**	**Period VI**	**Total**
Number of consultations	*N* = 7	*N* = 8	*N* = 9	*N* = 32	*N* = 20	*N* = 76
Core indicators	*n* (%)	*n* (%)	*n* (%)	*n* (%)	*n* (%)	
ESAS ^a^	5 (71.4)	4 (50.0)	2 (22.2)	4 (12.5)	1 (5.0)	
ECOG ^b^	7 (100)	6 (75.0)	8 (88.9)	24 (75.0)	18 (90.0)	
Weight	6 (85.7)	5 (62.5)	8 (88.9)	26 (81.2)	15 (75.0)	
Report sent to GP ^c^	1 (14.3)	8 (100)	9 (100)	26 (81.2)	17 (85.0)	
Documented core indicators ^e^	19 (67.9)	23 (71.9)	27 (75.0)	80 (62.5)	51 (63.8)	200
No. of core indicators for documentation ^d^	28	32	36	128	80	304
**Program fulfilment, first oncological consultations ^f^**						**65.8**
**Palliative care consultations**	**Period I**	**Period II**	**Period III**	**Period IV**	**Period VI**	**Total**
Number of consultations	*n* = 9	*n* = 11	*n* = 11	*n* = 38	*n* = 18	*N* = 87
Core indicators	*n* (%)	*n* (%)	*n* (%)	*n* (%)	*n* (%)	
ESAS ^a^	8 (88.9)	11 (100)	11 (100)	37 (97.3)	17 (94.4)	
ECOG ^b^	6 (66.7)	10 (90.9)	11 (100)	37 (97.3)	17 (94.4)	
Weight	8 (88.9)	10 (90.9)	11 (100)	36 (94.7)	17 (94.4)	
Report sent to GP ^c^	8 (88.9)	11 (100)	11 (100)	36 (94.7)	17 (94.4)	
Documented core indicators ^e^	30 (83.3)	42 (95.4)	44 (100.0)	146 (96.1)	68 (94.4)	330
No. of core indicators for documentation ^d^	*36*	*44*	*44*	*152*	*72*	*348*
**Program fulfilment, palliative care consultations ^f^**						**94.8%**
**Oncological consultations during chemotherapy.**	**Period I**	**Period II**	**Period III**	**Period IV**	**Period VI**	**Total**
Number of consultations	*n* = 31	*n* = 64	*n* = 53	*n* = 79	*n* = 45	*N* = 272
Core indicators	*n* (%)	*n* (%)	*n* (%)	*n* (%)	*n* (%)	
ESAS ^a^	25 (80.6)	53 (82.9)	38 (71.7)	3 (3.8)	4 (8.8)	
ECOG ^b^	22 (71.0)	54 (84.3)	39 (73.6)	58 (73.4)	43 (95.6)	
Weight	22 (71.0)	45 (70.3)	38 (71.7)	52 (65.9)	28 (62.2)	
Report sent to GP ^c^	10 (32.2)	64 (100)	52 (98.1)	67 (84.8)	36 (80.0)	
Documented core indicators ^e^	79 (63.7)	216 (84.3)	167 (78.8)	180 (57.0)	111 (61.7)	753
No. of core indicators for documentation ^d^	124	256	212	316	180	1088
**Program fulfilment, Oncological consultations during chemotherapy ^f^**						**69.20%**

^a^ Edmonton Symptom Assessment System [37]. ^b^ Eastern Cooperative Oncology Group performance status [38]. ^c^ GP: General practitioner. ^d^ The number of consultations within the period multiplied by 4 (number of required core indicators). ^e^ The actual number of documented indicators in the patient records. ^f^ Program fulfilment denotes the number of documented indicators, divided by the total number of required indicators, for each consultation type respectively.

## Data Availability

The datasets generated and/or analyzed during the current study are available from the corresponding author on reasonable request.
